# Materials with Negative Permittivity or Negative Permeability—Review, Electrodynamic Modelling, and Applications

**DOI:** 10.3390/ma18020423

**Published:** 2025-01-17

**Authors:** Jerzy Krupka

**Affiliations:** Institute of Microelectronics and Optoelectronics, Warsaw University of Technology, Koszykowa 75, 00-662 Warsaw, Poland; jerzy.krupka@pw.edu.pl or czar3k@o2.pl

**Keywords:** negative permittivity, negative permeability, magnetic plasmon, electric plasmon, lightning plasma ball

## Abstract

A review of natural materials that exhibit negative permittivity or permeability, including gaseous plasma, metals, superconductors, and ferromagnetic materials, is presented. It is shown that samples made of such materials can store large amount of the electric (magnetic) energy and create plasmonic resonators for certain values of permittivity, permeability, and dimensions. The electric and the magnetic plasmon resonances in spherical samples made of such materials are analyzed using rigorous electrodynamic methods, and the results of the analysis are compared to experimental data and to results obtained with other methods. The results of free oscillation and Mie scattering theories are compared. Similarities and differences between permittivity and permeability tensors for magnetized plasma and magnetized ferromagnetic materials are underlined. Several physical phenomena are explained on the grounds of rigorous electrodynamic analysis and experiments. These phenomena include unequal electric and magnetic energies stored in plasmonic resonators, the small influence of dielectric losses on the Q-factors of magnetic plasmon resonances, the role of radiation and dissipation losses on the properties of plasmonic resonators, and the theoretical possibility of the existence of lightning plasma balls.

## 1. Introduction

There are several natural materials that exhibit negative permittivity or negative permeability at certain frequency ranges. Among them are gaseous plasma in broad frequency ranges, metals and semiconductors at optical and infrared frequencies, and superconductors and ferromagnetic materials at microwave frequencies. The most extensive research on materials that exhibit negative permittivity has been undertaken for plasma. The fundamentals of plasma physics can be found in [1,2,3]. Many metals, e.g., gold, silver, and aluminum, exhibit negative permittivity values at optical frequencies, and nanoparticles that are made of them behave as electric plasmon resonators [4,5,6,7,8,9,10,11,12,13,14,15,16,17,18,19,20]. They have been employed in selective optical-induced heating, enhancement of non-linear optical excitation in nanomaterials, and the sensing of biological and chemical substances [8,9]. The main difference between ordinary resonators and plasmonic resonators is their size, with for plasmonic resonators being much smaller than the wavelength. Electric plasmon resonators have been observed in antient times and have been theoretically and experimentally studied for more than 100 years; however, so far, only a few papers have been published on the electrodynamic analysis of magnetic plasmon resonators [21,22,23,24]. Spherical yttrium iron garnet (YIG) resonators have been used at microwave and frequencies for a long time, but they have been considered as operating at ferromagnetic rather than plasmonic resonance. There are two analytic approaches to the electrodynamic analysis of plasmonic resonators. In the first one, the sample (resonator) is considered as an electromagnetic scatterer. For samples having sizes that are smaller than but comparable to the wavelength Mie [4], scattering theory is applicable. These days, Mie theory is well advanced [25], and publicly available computer programs allow computations of extinction $C_{ext}$ coefficients of spherical particles having arbitrary values of permittivity and permeability [26,27,28]. Mie extinction coefficient maxima correspond to the electromagnetic resonances excited in the sample by an incident plane wave. Mie scattering analysis is equivalent to analysis of forced oscillations of an electromagnetic resonator. In the second approach, free oscillations of a resonance object are considered. Formally, free oscillations are analyzed as solutions of a boundary value problem (eigenvalue problem) for source-free Maxwell equations. For low-loss resonators, resonance frequencies obtained with these two approaches perfectly agree, for both ordinary and plasmonic resonators [24]. Mie scattering theory is commonly used at optical frequencies because it directly corresponds to physical experiments performed at these frequencies with various types of spectrometers and microscopes. At microwave frequencies, the sizes of resonators are about four orders of magnitude larger than those in optical frequencies, which allows for precise machining of their shapes. In addition, vector network analyzers (VNAs) are available, which allow accurate measurements of the resonance frequencies, Q-factors, and coupling coefficients of resonance structures. For these reasons, the relative measurement accuracy of plasmonic resonator parameters at microwave frequencies is higher than at optical frequencies. 

The goal of this paper is to provide a review of materials that exhibit negative permittivity or permeability and a description of electrodynamic properties of the electric or the magnetic plasmon resonators that are made of them. We focus our attention on spherical resonators because analytic solutions of Maxwell equations are available for them and they create benchmarks for intercomparison studies. Although Mie scattering and free oscillation theories are already well established, less attention has been given in the literature to the analysis of electromagnetic energy storage and dissipation in dispersive media that exhibit negative permittivity or permeability. It has been already shown that for plasmonic resonators, the stored magnetic energy and the stored electric energy per period of oscillations are different, sometimes by orders of magnitude; however, this knowledge is still uncommon. Another problem that will be considered in this paper is the difference between permeability (permeability) for free and forced oscillation modelling. Complicated formulas related to electrodynamic analysis are avoided in this paper, but references for them are given.

## 2. Permittivity and Permeability in Dispersive Media

The complex permittivity ε and the complex permeability μ are material parameters that describe propagation of monochromatic electromagnetic waves but also the electromagnetic energy storage and dissipation in various media. Generally, permittivity and permeability are tensor quantities. In the description of material properties, the unitless relative permittivity $\varepsilon_{r}=\varepsilon /\varepsilon_{0}$ and the unitless relative permeability $\mu_{r}=\mu /\mu_{0}$ are used, where $\varepsilon_{0}$ and $\mu_{0}$ are the permittivity and permeability of a vacuum, respectively. If losses are present, permittivity (permeability) exhibits complex quantities: $\varepsilon_{r}=\varepsilon_{r}^{\prime }-j\varepsilon_{r}^{\prime \prime }$, ${\mu_{r}=\mu_{r}^{\prime }-j\mu }_{r}^{\prime \prime }$. We choose minus signs for the imaginary parts of permittivity and permeability, which is common at RF and microwave frequencies; however, in optics these signs are usually taken as a positive value. Consequently, the complex angular frequency for the free oscillations in our approach is taken as $\omega =\omega^{\prime }+j\omega^{\prime \prime }$ contrary to optics, where it is $\omega =\omega^{\prime }-j\omega^{\prime \prime }$. Conductivity σ can be formally included in the imaginary part of permittivity $\varepsilon_{r}=\varepsilon_{rd}-j\sigma /\left(\omega \varepsilon_{0}\right)$, where $\varepsilon_{rd}$ denotes the relative complex permittivity of medium excluding the conductivity term. The real parts of permittivity or/and permeability can be negative at some frequency ranges, but the electromagnetic energy stored in the areas of space filled with such media should be positive. General expressions for the electric and the magnetic energy densities in scalar dispersive media are as follows [29,30,31,32]:

(1) $$w_{e}=\frac{\partial (\omega \varepsilon_{r}^{\prime }(\omega ))}{\partial \omega }\varepsilon_{0}{\left|E\right|}^{2}$$

(2) $$w_{m}=\frac{\partial (\omega \mu_{r}^{\prime }(\omega ))}{\partial \omega }\mu_{0}{\left|H\right|}^{2}$$

For nondispersive media expressions, Equations (1) and (2) reduce to ${w_{e}=\varepsilon_{r}^{\prime }\left|E\right|}^{2}$ and ${w_{m}=\mu_{r}^{\prime }\left|H\right|}^{2}$, respectively. Dispersion relations for the naturally occurring passive media with negative permittivity (permeability) are such that Equations (1) and (2) are positive. Derivatives in Equations (1) and (2) can be considered as the “effective” permittivity (permeability) values of dispersive medium in the electromagnetic energy calculations. 

### 2.1. Models of Permittivity and Permeability 

Let us consider permittivity and permeability models for few natural media that exhibit negative permittivity or permeability at some frequency ranges. The most frequently used simple model of the complex permittivity is known as the Lorentz oscillator model, which is derived as a driven damped harmonic oscillator. According to this model, the complex permittivity can be written as follows:

(3) $$\varepsilon_{r}=1-\frac{\omega_{p}^{2}}{\omega^{2}-\omega_{0}^{2}-j\omega \Gamma }$$

where $\omega_{p}$—radian plasma frequency (rad/s), $\omega_{p}^{2}=N_{e}e^{2}/\varepsilon_{0}m_{e}$, $N_{e}$—free electron density, e—charge of the electron, $m_{e}$—the effective mass of the electron, Γ—damping factor (rad/s), and $\omega_{0}$—radian resonance frequency of bounded charges in solids involving the nucleus and the electron cloud.

The Lorentz model is usually used to describe the permittivity of dielectrics at optical frequencies and above. For gaseous plasma and conductors, where electrons are unbounded, the Drude [33,34] model of permittivity is used and can be considered as a simplification of the Lorentz model by assuming $\omega_{0}$ = 0. For metals, it is additionally assumed that permittivity at infinite frequency is not equal to unity but to $\varepsilon_{\infty }$. Thus, the relative permittivity of metals according to Drude model is expressed as follows.

(4) $$\varepsilon_{r}=\varepsilon_{\infty }-\frac{\omega_{p}^{2}}{\omega^{2}-j\omega \Gamma }$$

Electric plasma is a common state of matter in the universe. Free electron density in different plasmas that appear in the universe vary in the range of 20 decades or more from $10^{-5}\;{cm}^{-3}$ for interstellar and galactic plasmas to $10^{25}\;{cm}^{-3}$ at the center of stars like the Sun. For the ionosphere of the Earth, $N_{e}\sim 10^{10}\;{cm}^{-3}$, so $\omega_{p}\sim {6\times 10}^{6}\;rad/s$. The ionosphere reflects electromagnetic waves when permittivity becomes negative. For noble metals, $N_{e}\sim 10^{22}{\;cm}^{-3},$ so $\omega_{p}\sim 10^{16}\;rad/s$. 

The “effective” permittivity of $\partial (\omega \varepsilon_{r})/\partial \omega$ for the Lorentz and Drude models are positive across the whole frequency spectrum. 

When an ideal plasma is situated in an uniform external static magnetic field $B_{0}$ oriented along the z-axis of a Cartesian or cylindrical coordinate system, then its permittivity becomes a tensor quantity in Equation (5). In expressions for tensor components, we used notation adapted from [35].

(5) $$\overset{̿}{\varepsilon }=\mu_{0}\left[\begin{array}{ccc} \varepsilon_{⊥} & jg & 0\\ -jg & \varepsilon_{⊥} & 0\\ 0 & 0 & \varepsilon_{\left|\right|} \end{array}\right]$$

$$\varepsilon_{⊥}=1-\frac{\omega_{p}^{2}}{\omega^{2}-\omega_{B}^{2}},\;g=\frac{\omega_{p}^{2}\omega_{B}}{\omega (\omega^{2}-\omega_{B}^{2})},\;\varepsilon_{\left|\right|}=1-\frac{\omega_{p}^{2}}{\omega^{2}}$$

$$\omega_{B}=\frac{e}{m_{e}}B_{0}=\gamma_{e}B_{0},\;{\gamma_{e}=e/m}_{e}=1.75882\times 10^{11}\;\mathrm{rad}/s/T$$

The permittivity of plasma in a static magnetic field for circular polarization of the electric field in the plane orthogonal to the static magnetic field is given by the following expressions.

(6) $$\varepsilon_{r+}=\;\varepsilon_{⊥}+g\;=1-\frac{\omega_{p}^{2}}{\omega (\omega -\omega_{B})}$$

(7) $$\varepsilon_{r-}=\;\varepsilon_{⊥}-g\;=1-\frac{\omega_{p}^{2}}{\omega (\omega +\omega_{B})}$$

Here, $\varepsilon_{r-}$ corresponds to the left-handed circular polarization, while $\varepsilon_{r+}$ corresponds to the right-handed circular polarization. The “effective” permittivity values for the circular polarization in the direction perpendicular to the static magnetic field are positive for arbitrary frequency values.

(8) $$\frac{\partial \left(\omega \varepsilon_{r+}\right)}{\partial \omega }=1+\frac{\omega_{p}^{2}}{{\left(\omega -\omega_{B}\right)}^{2}}$$

Magnetic properties of ferromagnetic materials in the presence of an internal static magnetic field $H_{0}$ are described by permeability tensor (Polder tensor), given in Equation (9) [36]. Similarly, as for plasma, it is assumed that $H_{0}$ is oriented along the z-axis of a Cartesian or cylindrical coordinate system.

(9) $$\overset{̿}{\mu }=\mu_{0}\left[\begin{array}{ccc} \mu & j\kappa & 0\\ -j\kappa & \mu & 0\\ 0 & 0 & \mu_{\left|\right|} \end{array}\right]$$

where $\mu =1+\frac{H_{0r}+j\alpha w}{H_{0r}^{2}–{(1+\alpha^{2})w}^{2}+2j{\alpha H}_{0r}w}$, $\kappa =\frac{w}{H_{0r}^{2}–{(1+\alpha^{2})w}^{2}+2j{\alpha H}_{0r}w}$, $H_{0r}=H_{0}/M_{S}\;$, ${w=f/f}_{m}$, f is frequency, $f_{m}=\gamma M_{S}$ (often named as the natural frequency of ferromagnetic resonance), $H_{0}$—the internal static magnetic field in ferromagnetic medium (kA/m), $M_{S}$—saturation magnetization in (kA/m), α—Gilbert damping factor, and γ—gyromagnetic ratio γ ≈ 35.217 MHz/(kA/m). For saturated medium, when $H_{0}>M_{S}$, $\mu_{\left|\right|}\;$= 1.

For circularly polarized electromagnetic fields (in the plane that is orthogonal to the static magnetic field), the permeability of the ferromagnetic material can be represented by two scalar quantities corresponding to the left-handed circular polarization (sign “−”), and the to the right-handed circular polarization (sign“+”).

(10) $$\mu_{r\pm }=\mu \pm \;\kappa$$

Permeability $\mu_{r+}$ exhibits a resonance character and is negative for some static biasing fields, while permeability $\mu_{r-}$ is non-resonance and positive. For magnetically lossless medium, the following is obtained:

(11) $$\mu_{r+}=1+\frac{1}{H_{0r}-w}$$

Let $\omega_{m}=2\pi f_{m}$ and $\omega_{H}=2\pi \gamma H_{0}$ (in a vacuum, $\omega_{H}=\omega_{B}$; however, for ferromagnetic materials, these values are slightly different because γ is slightly different from $\gamma_{e}$). Then, the expression for $\mu_{r+}$ can be written as follows:

(12) $$\mu_{r+}=1+\frac{\omega_{m}}{\omega_{H}-\omega }$$

The “effective” permeability for $\mu_{r+}$ is expressed as follows:

(13) $$\frac{\partial \left(\omega \mu_{r+}\right)}{\partial \omega }=1+\frac{H_{0r}}{{\left(H_{0r}-w\right)}^{2}}=1+\frac{\omega_{m}\omega_{H}}{{\left(\omega_{H}-\omega \right)}^{2}}$$

Similarly, the “effective” permittivity for $\varepsilon_{r+}$ in plasma, when subjected to the static magnetic field, shows that the “effective” permeability for $\mu_{r+}$ is positive. Further similarities between expressions for $\varepsilon_{r+}$ and $\mu_{r+}$ should be noted. The term $\omega_{p}^{2}/\omega \;$ in Equation (6) corresponds to $\omega_{m}$ in Equation (12). For known ferromagnetic materials, $\omega_{m}$ lies in the microwave frequency range, while $\omega_{p}^{2}/\omega$ for metals belongs to optical frequency range. The influence of the static magnetic field on $\varepsilon_{r+}$ in metals at optical frequencies is negligibly small for the fields available in laboratories because $\omega_{B}\ll \omega$. However, the static magnetic field is commonly used for frequency tuning of magnetic plasmon resonators.

Conductivity of a superconductor at temperatures $T<T_{c}$ can be described by the two-fluid conductivity model [37]. For an isotropic superconductor, its conductivity is given as $\sigma \left(\omega \right)=\sigma_{1}-j\sigma_{2}$, where $\sigma_{1}$ and $\sigma_{2}$ are conductivities related to normal and superconducting charge carriers, respectively. Here, σ_1_ is responsible for the normal conduction, and σ_2_ is responsible for the kinetic inductance. Substituting the complex conductivity into the expression for the complex permittivity, one obtains the following: $\varepsilon_{r}=\varepsilon_{\infty }-\sigma_{2}/\left(\omega \varepsilon_{0}\right)-j\sigma_{1}/\left(\omega \varepsilon_{0}\right)$. Electromagnetic fields in superconductors in both the superconducting and the normal state decay exponentially in the direction of propagation. For a lossless superconductor (when $\sigma_{1}=0$) the electromagnetic energy is stored without dissipation in the superconducting medium. The distance when an evanescent electromagnetic fields decays to 1/*e* is called the penetration depth λ ($\lambda^{2}=1/\left(\omega \mu_{0}\sigma_{2}\right)$. If $\sigma_{2}\gg \sigma_{1}$, then $\varepsilon_{r}$ can be written as $\varepsilon_{r}\approx -c^{2}/{(\omega }^{2}\lambda^{2})$. Then, the resulting “effective” permittivity responsible for the electric energy storage in superconductor is $\partial (\omega \varepsilon_{r})/\partial \omega =c^{2}/{(\omega }^{2}\lambda^{2})$. For high temperature superconductors such as YBCO, the penetration depth is on the order of 250 nm. Thus, at a frequency of 10 GHz, the “effective” permittivity is on the order of ${3.7\times 10}^{8}$. This means that a very large electric energy density is stored in the superconductor, even at microwave frequencies; however, the energy is stored in a small volume and restricted by the penetration depth. At lower frequencies, the electric energy density would become even larger because it is proportional to $\omega^{-2}$. The transition from the superconducting state to the normal state, due to the change of temperature, results in very rapid electromagnetic energy release, called a quench, which is occasionally observed in superconducting magnets and in particle accelerators. 

### 2.2. Experimental Data for Some Materials with Negative Permittivity or Permeability

The largest amount of experiments on materials that exhibit negative permittivity values have been performed for noble metals at optical frequencies [11,12,13,14,15], and they will not be reproduced here. Good fit to the experimental data [15] for gold in visible and near infrared frequencies has been obtained using the modified Drude model [18] with the following parameters: $\varepsilon_{\infty }=9.84,\;f_{p}=2.179\times {\;10}^{15}\;$ Hz, and $\Gamma =1.74\times {\;10}^{13}$ Hz. 

**Figure 1:**
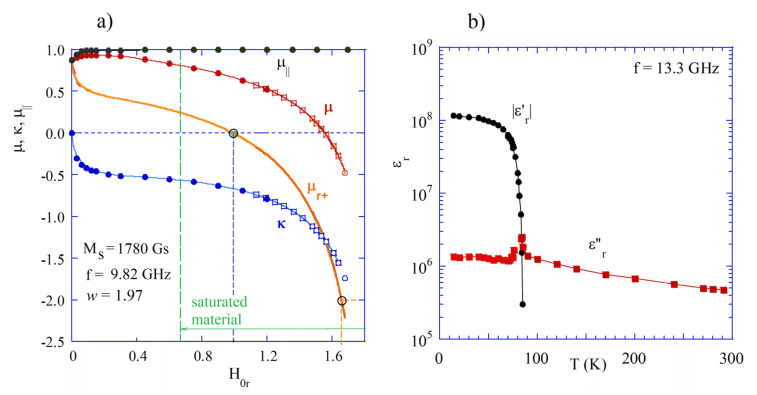
(**a**) Measured permeability tensor components for thin polycrystalline YIG rods versus a normalized static magnetic field (data used for the preparation of this figure were taken from [38,39]). (**b**) The real and the imaginary part of permittivity for thin YBCO films versus temperature at 13.3 GHz (based on experiments reported in [40]). © IEEE Publishing. Reproduced from [40] with permission. All rights reserved.

In Figure 1, experimental data are presented for the permeability of YIG 40 and permittivity of YBCO, which become negative at microwave frequencies. As it is seen in Figure 1a, both the diagonal component of the permeability tensor μ and permittivity $\mu_{r+}$ corresponding to the right-handed circular polarization become negative when the static magnetic field is sufficiently large, but still smaller than the field corresponding to the ferromagnetic resonance when $H_{0r}=w$. By definition, in the lossless case, permittivity components μ and κ become singular at the ferromagnetic resonance. It should be noted that measured permeability values perfectly agree with the Polder tensor model for saturated materials. 

In Figure 1b, the permittivity components of thin YBCO films versus temperature are presented and scaled to 13.3 GHz. The data shown in this figure are based on measurements of the complex conductivity components in [40] and formula $\varepsilon_{r}\approx -\sigma_{2}/\left(\omega \varepsilon_{0}\right)-j\sigma_{1}/\left(\omega \varepsilon_{0}\right)$. The real part of the permittivity of YBCO is negative at cryogenic temperatures, up to its critical temperature (about 86 K) where superconductivity disappears. It should be noted that the real part of permittivity is negative ($\varepsilon_{r}^{\prime }\approx -{1.1\times 10}^{8}$). Its modulus is extremely large and about two orders of magnitude larger than the imaginary part of permittivity.

## 3. Free and Driven Electromagnetic Oscillations in Resonance Structures Containing Dispersive Materials

**Figure 2:**
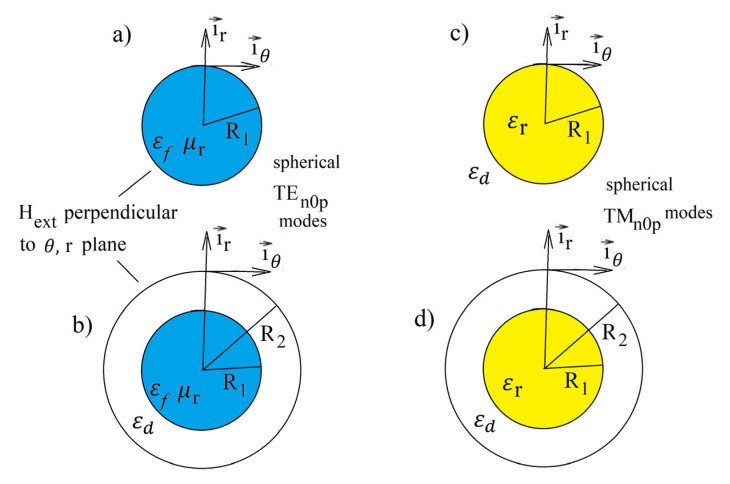
(**a**) A spherical gyromagnetic sample in free space, (**b**) a spherical gyromagnetic sample in a perfectly spherical conductor enclosure, (**c**) a spherical electric plasmon sample in free space, and (**d**) a spherical electric plasmon sample in a perfectly spherical conductor enclosure.

The electromagnetic response of an isolated plasmonic resonator where a plasmon is excited by monochromatic signal is characterized by the model of a driven damped oscillator. Free oscillations describe harmonic, exponentially decaying electromagnetic fields in a resonance system. In electrodynamic approaches, free oscillations are formulated as a boundary value problem for Maxwell’s equations. Solutions of the eigenvalue problem lead to the determination of the electromagnetic field distribution, the resonance frequencies, and the Q-factors in the resonance structure. The resonance frequencies ${\hat{\omega }}_{i}$ are complex ${\hat{\omega }}_{i}=\omega_{i}^{\prime }+j\omega_{i}^{\prime \prime }$ and describe the time dependence of the electromagnetic fields for the *i*-th free evanescent harmonic oscillation. The real part of the complex frequency describes the oscillatory term, while the imaginary part describes the evanescent term. Analytic solutions of the eigenvalue problem related to free oscillations can be obtained for resonance structures that possess certain kinds of symmetry with nonuniformity of material properties along only one coordinate of an orthogonal coordinate system, e.g., Cartesian, cylindrical, or spherical. In this paper, we focus our attention on the structures shown in Figure 2. Figure 2a,b corresponds to the gyromagnetic medium with permeability $\mu_{r+}$ in Equation (10) in the *r*-θ plane, while Figure 2c,d corresponds to the medium described by permittivity $\varepsilon_{r+}$ in Equation (6) in this plane. Rigorous solutions of Maxwell’s equations for these structures are only available for isotropic media; however, these solutions remain valid for some modes in gyromagnetic medium. For the ${TE}_{n0p}$ modes in a spherical coordinate system (subsequent mode subscripts correspond to θ,φ,r coordinates), the magnetic field has two components $H_{r}$ and $H_{\theta }$, and the electric field has only one component $E_{\varphi }$. If we chose a circularly polarized RF magnetic field in the *r*- θ plane that is perpendicular to the static magnetic field, then the RF field will “see” only the permeability $\mu_{r\pm }$ in this plane. Because the electromagnetic fields of ${TE}_{n0p}$ modes do not depend on the coordinate φ, the electromagnetic field distribution will be the same as that for the isotropic case. 

The validity of such an approach for the analysis of spherical ${TE}_{n0p}$ modes in a spherical gyromagnetic sample has been supported by experiments [21,22], FE numerical simulations [41], and the magnetostatic model [42]. When the diameter of the sphere converges to zero, then the electrodynamic solutions of the electrodynamic eigenvalue problem converge to the magnetostatic ones. The same arguments hold for the ${TM}_{n0p}$ modes in the structures shown in Figure 2c,d. 

Let us focus our attention on the ${TE}_{n0p}$ modes in gyromagnetic medium. For the structures shown in Figure 2a,b, the complex resonance frequencies of the ${TE}_{n0p}$ modes can be found as the complex roots ${\hat{\omega }}_{i}$, i=(n,p) of transcendental equations (TDE), which have a general form $F\left(\hat{\omega },\mu_{r}\left(\hat{\omega }\right)\right)=0$. By determining ${\hat{\omega }}_{i}$, one can also uniquely determine $\mu_{r}\left({\hat{\omega }}_{i}\right)$. The approximate values of resonance frequencies for infinitesimally small gyromagnetic samples can be found using a magnetostatic model [42]. The dominant mode in the magnetostatic approximation (the mode of uniform precession) for the spherical sample has only two magnetic field components in the plane, which is perpendicular to the static magnetic field. This mode corresponds to the spherical ${TE}_{101}$ electrodynamic mode (magnetic dipole mode). Such correspondence is also valid for higher-order spherical ${TE}_{n0p}$ modes (multipole magnetic modes), which have only two magnetic field components in the plane perpendicular to the static magnetic field. However, due to the anisotropy of permeability, the resonance frequencies of ${TE}_{nmp}$ modes with *m* > 0 are different than those for the ${TE}_{n0p}$ modes in the isotropic medium. The resonance frequencies of such modes and all quasi ${TM}_{nmp}$ modes can only be found using advanced electrodynamic numerical simulations, e.g., the finite element method. 

In the formulation of the eigenvalue problem for lossy resonance structures, the frequency has to be complex. It is should be underlined that the complex frequency has to be included in definitions of permittivity and permeability. The unloaded Q-factor (Q-factor excluding coupling losses) of *i*-th oscillations can be determined as $Q_{ui}=Re({\hat{\omega }}_{i})/\left(2Im\left({\hat{\omega }}_{i}\right)\right)$. For low-loss, non-radiating resonance structures, a simplified method of Q-factor determination is often employed. In the simplified method, in the first step of analysis, the system is assumed to be lossless. In such cases, we have steady state harmonic oscillations, and the resonance frequencies are real. In the next step, the Q-factors are computed according to Formula (14).

(14) $$\begin{array}{l} Q_{ui}=2\pi \frac{average\;energy\;stored\;duringa\;cycle}{average\;energy\;dissipated\;per\;cycle}\\ =\frac{\frac{1}{4}\left\{{∭}_{V}\varepsilon_{0}{\frac{\partial \left[{\omega \varepsilon }_{r}^{\prime }\left(\omega \right)\right]}{\partial \omega }}_{|\omega =\omega_{i}}{{|E}_{i}|}^{2}dv+{∭}_{V}\mu_{0}{\frac{\partial \left[\omega \mu_{r}^{\prime }(\omega )\right]}{\partial \omega }}_{|\omega =\omega_{i}}{\left|H_{i}\right|}^{2}dv\right\}}{\frac{1}{2}\left({∭}_{V}\varepsilon_{0}{\varepsilon_{r}^{\prime \prime }\left(\omega_{i}\right)\left|E_{i}\right|}^{2}dv+\mu_{0}\mu_{r}^{\prime \prime }(\omega_{i}){∭}_{V}{\left|H_{i}\right|}^{2}dv\right)} \end{array}$$

Integration in the numerator of Equation (14) is performed over the whole volume of the resonance structure, including the exterior part of the structure for open resonators (Figure 2a,c). 

**Figure 3:**
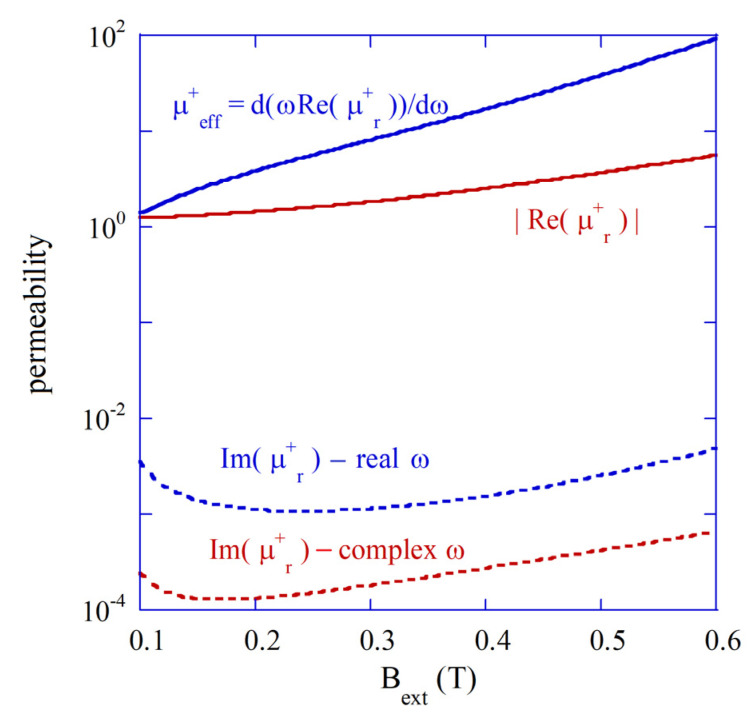
Components of permeabilities for transient (complex $\hat{\omega }$) and steady state solutions (real w) for the shielded plasmonic ${TE}_{101}$ mode and a single-crystal YIG sphere with $\varepsilon_{f}=16$, $M_{S}=140\frac{kA}{m},$ $R_{2}=4.4\;mm{,\;and\;R}_{1}=2.5\;mm$ in a spherical metal enclosure (TDE as in [22,23]).

In Figure 3, the modulus of the real part of permeability $\left|\mu_{r+}\right|$, the “effective” permeability $\partial \left(\omega \mu_{r+}\right)/\partial \omega$, and the imaginary parts of permeability for the real and the complex ω are presented. The imaginary part of the permeability for the complex frequency (for free damped oscillations) is of an order of magnitude smaller than that for the real frequency (steady state solutions). The real part of permeability is negative, and its modulus is up to one order of magnitude smaller than the “effective” permeability. However, as it has been shown in [22], Q-factor values computed with the two approaches remain essentially the same. The steady state solution approach provided in Equation (14) allows for the direct determination of the stored electric and magnetic energies per the cycle of oscillation.

## 4. Computations of Resonance Frequencies and Q-Factors of Spherical Samples Made of Materials with Negative Permittivity or Negative Permeability Values

**Figure 4:**
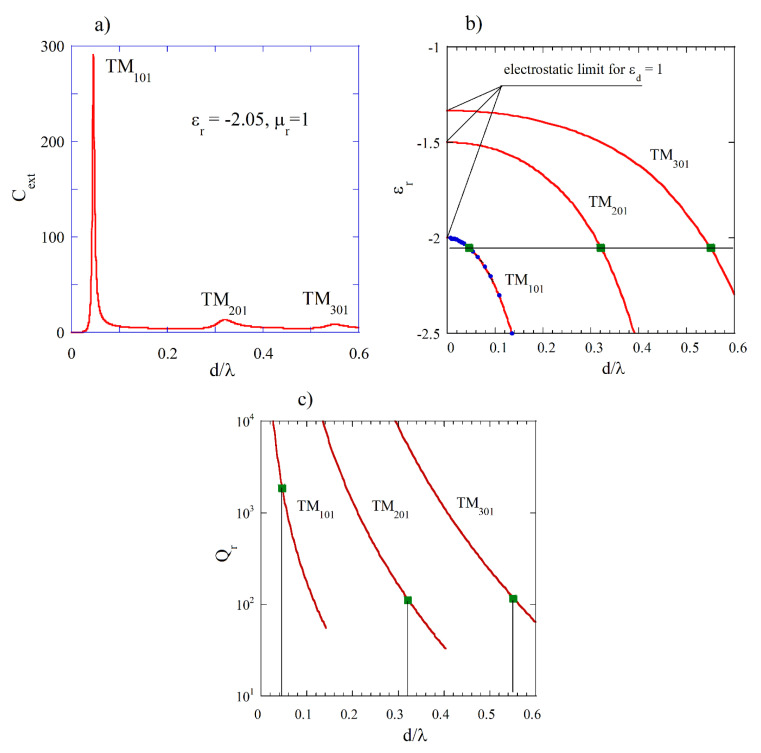
(**a**) Computed Mie spectrum of the extinction coefficient $C_{ext}$ for a low-loss nondispersive sphere with $\varepsilon_{r}=-2.05$ and $\mu_{r}=1$ using programs from [26]. (**b**) Permittivities corresponding to the three plasmon modes versus normalized resonance frequencies d/λ (TDE solutions for $\varepsilon_{\infty }=1,\;\Gamma =0$, and $\varepsilon_{d}=1$). Solid green squares correspond to three normalized frequencies evaluated from Mie spectrum ($C_{ext\_max})$ in Figure 4a; solid blue circles represent values evaluated from Mie spectra obtained for various $\varepsilon_{r}$. (**c**) Q-factors due to radiation losses ($Q_{r}$) for lossless medium (TDE solutions). Green squares represent $Q_{r}$ values corresponding to $\varepsilon_{r}=-2.05$.

Complex resonance frequencies of free oscillations for the ${TM}_{nmp}$ modes (subsequent subscripts indicate the mode orders along θ, φ,and r spherical coordinates, respectively) in an isotropic sphere having diameter *d* and permittivity as noted in Equation (4) can be found as solutions of appropriate transcendental equation (TDE) involving spherical Bessel functions [18,24]. The resonance frequencies of spherical modes in isotropic medium are *n*-fold degenerated (they do not depend on the azimuthal mode number *m*). Alternatively, the resonance frequencies of the ${TM}_{nmp}$ modes can be evaluated from the geometric parameter values ($x=kR_{1}=\omega R_{1}/c=\pi d/\lambda$) corresponding to maxima ($C_{ext\_max})$ of the extinction coefficient $C_{ext}$ function using Mie scattering computer programs. In Figure 4a, the computed Mie spectrum of extinction $C_{ext}$ coefficient for $\varepsilon_{r}=-2.05$ and $\mu_{r}=1$ is presented where three maxima corresponding to the three ${TM}_{nm1}$ modes with *n* = 1, 2, and 3 can be observed. In Figure 4b, the permittivity values corresponding to the three plasmon modes versus normalized resonance frequencies (TDE solutions for $\varepsilon_{\infty }=1,\;\Gamma =0$, $\varepsilon_{d}=1$) are presented. Solid green squares denote three normalized frequencies evaluated from Mie spectrum shown in Figure 4a, and solid blue circles show normalized frequencies evaluated from Mie spectra obtained for various $\varepsilon_{r}$. The red shift of plasmonic resonances with the increase in the diameter of the sphere, observed in experiments with gold particles at optical frequencies [20], corresponds to the decrease in permittivity required for the appearance of these resonances, as d/λ increases. In Figure 4c, Q-factors due to radiation losses ($Q_{r}$) for lossless medium (TDE solutions) are presented. Green squares represent $Q_{r}$ values corresponding to $\varepsilon_{r}=-2.05$. For d/λ converging to zero, permittivity values related to the appearance of plasmonic resonances converge to the electrostatic approximations (Rayleigh scattering) given by the expression: $\varepsilon_{r}/\varepsilon_{d}=1-\left(2n+1\right)/n$. As expected, from the theory of spherical dipole antenna radiation, Q-factors due to radiation losses decrease with the increase in d/λ as $Q_{r\;}\propto \;{\left(d/\lambda \right)}^{-3}$ (Figure 4c). Low peak values of $C_{ext}$ for ${TM}_{201}$ and ${TM}_{301}$ modes in Figure 4a are related to the relatively low $Q_{r\;}$ values for these modes. It should be noted that for a fixed d/λ value, the $Q_{r\;}$ values increase with the increase in the mode index n. 

**Figure 5:**
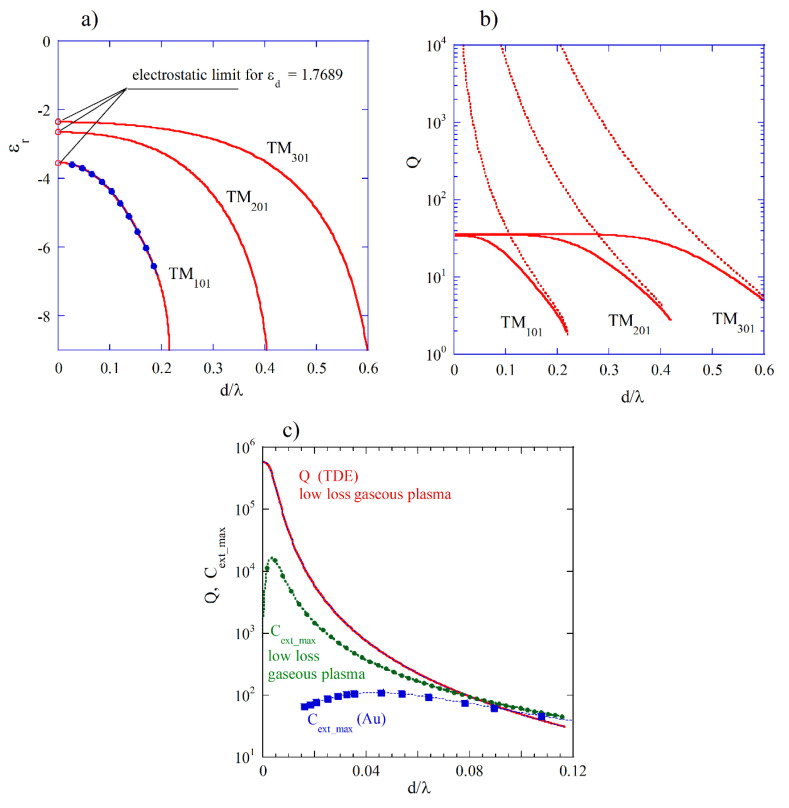
(**a**) Permittivity values corresponding to the three plasmon modes (TDE solutions) for gold spheres immersed in water assuming $\varepsilon_{\infty }=9.84,\;f_{p}=2.179\times 10^{15}\;$ Hz, $\Gamma =1.74\times {\;10}^{13}$ Hz, and $\varepsilon_{d}=1.7689$. Solid blue circles represent the results obtained from Mie spectra. (**b**) Q-factors of three plasmonic resonances. Solid lines denote the total Q-factors computed for gold spheres, and broken lines represent the Q-factors due to radiation losses (lossless medium, TDE solutions). (**c**) Mie extinction coefficients $C_{ext\_max}$ for gold (blue squares); $C_{ext\_max}$ for an hypothetical low-loss gaseous plasma (green circles) with $\varepsilon_{\infty }=1,\;f_{p}=10\;$ MHz, $\varepsilon_{d}=1$, and $\Gamma =f_{p}\times {\;10}^{-6}$; and the total Q-factor for the low-loss gaseous plasma (TDE solutions).

In Figure 5a, the permittivity values corresponding to the three plasmon modes (TDE solutions) of gold spheres immersed in water are presented assuming $\varepsilon_{\infty }=9.84,\;f_{p}=2.179\times 10^{15}\;$ Hz, $\Gamma =1.74\times 10^{13}$ Hz, and $\varepsilon_{d}=1.7689$. Solid blue circles represent results evaluated from Mie spectra for $\varepsilon_{r}$ values obtained from TDE solutions. In Figure 5b, Q-factors of the three plasmonic resonances are presented (TDE solutions). Broken lines represent Q-factors due to radiation losses ($Q_{r}$) computed for Γ=0. As it is seen, for the ${TM}_{101}$ mode, radiation losses become dominant when d/λ>0.1. The results of computations shown in Figure 5a,b correspond to the experimental data shown in [20]. In Figure 5c, the results of computations of extinction coefficient maxima ($C_{ext\_max})$ are presented (using programs reported in [26]) for gold and for a hypothetical low-loss gaseous plasma ($\varepsilon_{\infty }=1,\;f_{p}=10\;$ MHz, $\varepsilon_{d}=1$, $\Gamma =f_{p\;}\times {\;10}^{-6}$). Additionally, Q-factor values obtained for the low-loss plasma from the TDE solutions are shown. As it is shown in Figure 5c, Mie extinction coefficient maxima ($C_{ext\_max})$ approach the largest values near the points when the absorption and the radiation losses become equal to each other for both low-loss plasma and gold. Such behavior is related to the variations in the coupling rates between the plasmonic resonator and the source of electromagnetic energy (in Mie theory, the plane electromagnetic wave). The coupling rate is related to the $Q_{r}$ value. The smaller the $Q_{r}$ value is, the larger the coupling rate. Critical coupling of the resonator enables the efficient transfer of energy from a source of the EM energy to the resonator. Such a condition is achieved when the total loss rate of the resonator (including radiation and absorption losses) is equal to the coupling rate. At RF and microwave frequencies, coupling between a resonator and a waveguide can be achieved by many different methods, and accurate adjustment and measurements of coupling coefficients are possible [43].

Gaseous plasma balls are occasionally observed in nature as lightning balls [44]. Assuming parameters of gaseous plasma in our modelling and uniform electron density distribution within the ball, we have the largest value of $C_{ext\_max}$ for $d/\lambda \approx 3.5\times 10^{-3}$ (Figure 5c). In such a case, the resonance frequency of the ${TM}_{101}$ mode corresponds to Rayleigh scattering, so ${f\approx f}_{p}/\sqrt{3}$ ≈ 5.77 MHz (λ≈52 m). Therefore, the diameter of the ball corresponding to the largest value of $C_{ext\_max}$ is about 18 cm, and the electromagnetic energy transfer to the ball at a frequency of 5.77 MHz is the most efficient. If the density of electrons in the plasma ball becomes smaller due to the recombination of electrons and nuclei of plasma, then $f_{p}$ will be shifted toward lower frequencies. In such a case, the resonance frequency of the ${TM}_{101}$ mode will be smaller as well. Simulataneously, the Q-factor due to radiation losses will be much larger because $Q_{r\;}\propto \;{\left(d/\lambda \right)}^{-3}$, and the electromagnetic losses within the ball will be predominantly caused by the absorption. A review of measurements of the RF spectrum of radiation from lightning [45] has shown that the dominant part of the RF spectrum belongs to low acoustic and sub-acoustic frequencies. The lower frequency is, the longer the lifetime of oscillations. As an estimate of the lifetime (when the magnitude of the electric field decreases by a factor of *e*), we can use the expression τ~2Q/ω. Assuming $\omega =10^{3}$ rad/s (acoustic frequency) and $Q=10^{5}$, one obtains τ=200s. When the free electron density in plasma becomes smaller, the plasma frequency and the resonance frequency of the ${TM}_{101}$ mode are shifted to the lower frequencies, and the lifetime becomes longer. The following question remains: Howe has the lightning plasma ball been created at some initial state? Nonetheless, the existence of a lightning plasma ball as a plasmonic resonance with a relatively long lifetime has been theoretically confirmed.

**Figure 6:**
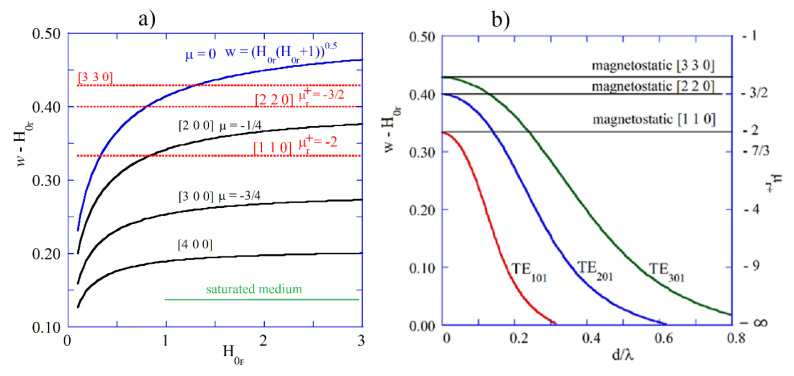
(**a**) $w-H_{0r}$ for few modes in magnetostatic approximation computed from Fletcher’s TDE [42]. (**b**) $w-H_{0r}$ as function of d/λ (*d* = 0.5 mm, $\varepsilon_{f}=16$, $M_{S}=140\;kA/m$) computed as solutions of TDE [23] for the first three ${TE}_{n01}$ magnetic plasmon modes of spherical sample (Figure 2a).

Figure 6a shows $w-H_{0r}$ (corresponding to $\omega_{H}-\omega$) versus normalized internal static magnetic field ($H_{0r}=H_{0}/M_{S}$) for a few modes in the magnetostatic approximation computed from Fletcher’s TDE [42]. Having known w  and $H_{0r}$, one can determine $\mu_{r+}$ from Equation (11) and $\mu =1+1/(H_{0r}^{2}-w^{2})$ for a lossless medium. For the most of these “magnetostatic” modes, the corresponding $\mu_{r+}$ and μ exhibit negative values. In Figure 6b, graphs of $w-H_{0r}$ versus d/λ are presented for the electrodynamic ${TE}_{101}$, ${TE}_{201}$, and ${TE}_{301}$ modes computed as solutions of TDE 23 for the structure shown in Figure 2a. The right vertical axis represents $\mu_{r+}$, corresponding to the $w-H_{0r}$ values. For infinitesimally small samples, electrodynamic solutions converge to the magnetostatic ones, similar to that noted for the electric plasmon modes. Corresponding permeability values for infinitesimally small samples are given by the expression $\mu_{r+}=1-\left(2n+1\right)/n$. In the electrodynamic approach for large samples, $\mu_{r+}$ values corresponding to magnetic plasmonic resonances converge to minus infinity (ferromagnetic resonance in an infinite medium). The same is true for $\varepsilon_{r}$ and $\varepsilon_{r+}$ values corresponding to electric plasmonic resonances. 

**Figure 7:**
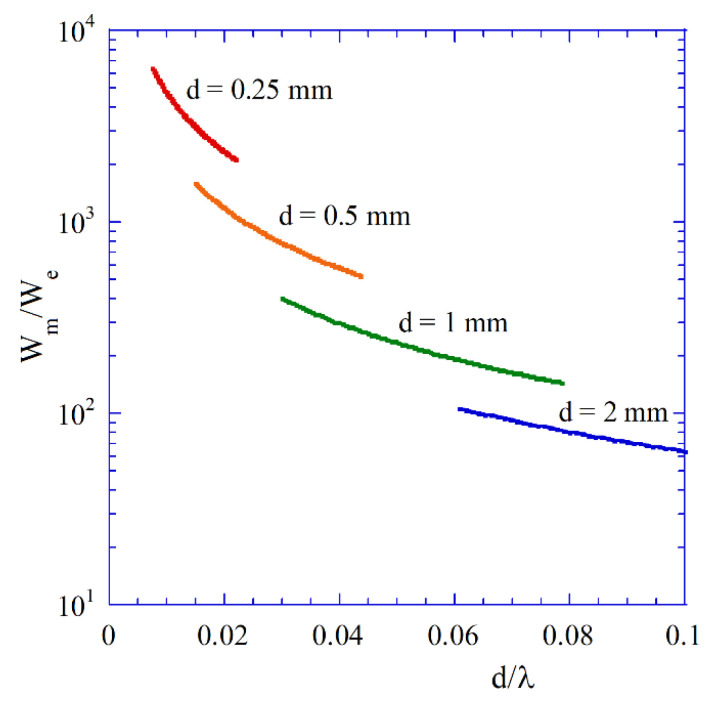
Ratios of the magnetic to the electric energy stored per period of oscillations versus d/λ for the ${TE}_{101}$ modes in lossless spherical samples having different diameters, for $\varepsilon_{f}=16$, $M_{S}=140\;kA/m$, and a structure as shown in Figure 2b with $R_{2}=10R_{1}$. Computations of energies using expressions from Equation (14).

Figure 7 shows the ratios of the magnetic to the electric energies stored per period of oscillations versus d/λ for the ${TE}_{101}$ modes in lossless spherical samples having different diameters as well as $\varepsilon_{f}=16$ and $M_{S}=140\;kA/m$. Variations of d/λ for fixed diameter of samples have been achieved by the change in the external static magnetic field in the expression for permeability $\mu_{r+}$ (12). Computations of energies have been performed by obtaining magnetic and electric field distributions for a lossless sample (solutions of TDE [23]) and using expressions for the energies in the numerator of Equation (14). The magnetic to the electric energy ratio varies by several orders of magnitude. For d/λ converging to zero, the ratio is converging to infinity, and sample behaves as pure magnetic dipole, as expected from the magnetostatic model. For very small samples, the influence of dielectric losses, including conductor losses, become negligibly small. As it has been shown in [38], for ferromagnetic metal thin films having a thickness of 50 nm and conductivity $\sigma =5\times 10^{6}$ S/m, such large dielectric losses (with conductivity contributing to the imaginary part of permittivity) cause the Q-factor of the plasmonic resonance to change by only a few percent. From the electrodynamic point of view, thin ferromagnetic films also creates plasmonic resonators [38].

**Figure 8:**
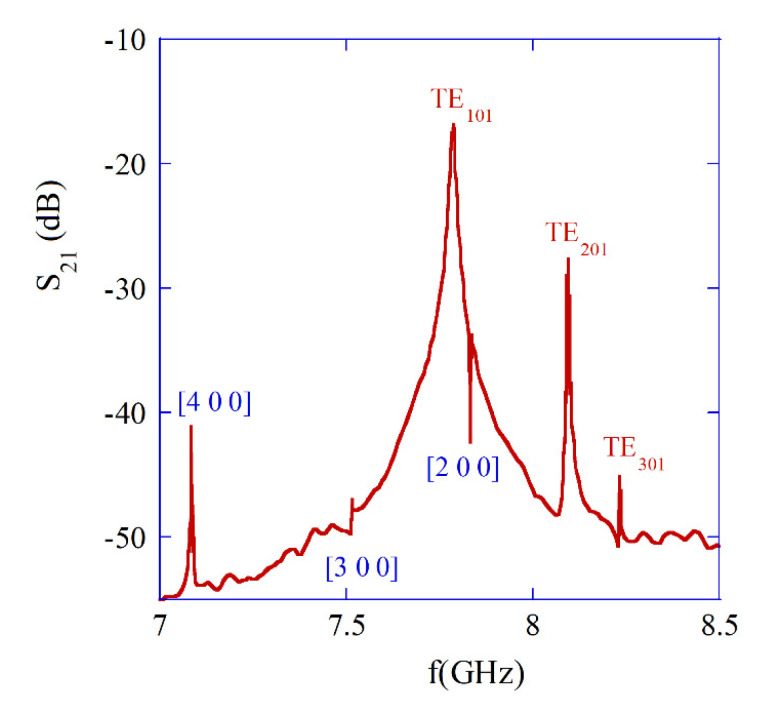
Measured |S21| values of a sub-wavelength metal resonator containing a single-crystal YIG sample (*d* = 0.5 mm, ΔH = 0.47 Oe at 10 GHz) for $B_{ext}\approx 0.278\;T$ ($H_{0r}\approx 1.22)$. Details of experiment are noted in [38] but with a different $B_{ext}$.

In Figure 8, measurement results are presented, showing that a few resonance frequencies for the single-crystal YIG sample with a diameter of 0.5 mm [38]. Three plasmonic ${TE}_{101}$, ${TE}_{201}$, and ${TE}_{301}$ modes are clearly seen. Measurements have been performed using VNAs. In the frequency range shown in Figure 8, d/λ<0.014, so frequencies obtained with magnetostatic approximation agree well with the electrodynamic ones. In addition, frequency separations between subsequent ${TE}_{n01}$ modes with n = 1, 2, and 3 are similar for both the electrodynamic and the magnetostatic approaches. 

The remaining resonances seen in Figure 8 correspond to the magnetostatic modes [4 0 0], [3 0 0], and [2 0 0], respectively (compare Figure 6a). As it has been mentioned, in the electrodynamic approach, for gyromagnetic medium, the ${TE}_{nm1}$ modes with *m* > 0, but also quasi ${TM}_{nm1}$ modes are hybrid, and advanced numerical methods have to be used for their analysis, e.g., the finite element method [41,46]. This is outside the scope of this paper since these modes are usually undesirable in practice. Magnetic plasmon resonators used in tunable microwave filters have Q-factors on the order of 10,000, which are predominantly related to the Gilbert damping factor Q≈1/(2α) of ferrite material [47].

## 5. Applications

As it has been already mentioned, materials with negative permeability values have found practical applications in the construction of various microwave magnetically tunable devices, such as filters, oscillators, and circulators. Resonances observed in thin ferromagnetic (or ferrimagnetic) films exhibit plasmonic nature [38]. Such films can also support propagation of spin waves. 

In DC and RF applications, superconductors are used for the construction of strong electromagnets and superconducting quantum interference devices such as SQUIDs or Josephson voltage standards. At microwave frequencies, superconductors are used in the realization of continuous-wave linear accelerators and for the fabrication of extremely high Q-factor microwave resonators.

Research on electric plasmon resonators is rapidly growing at infrared, optical, and ultraviolet frequencies. New plasmonic materials are studied, such as semiconductors [48] or metamaterials, and they are applied in the construction of optical devices such as thermally tunable biosensors [49,50], antennas [51] or photon-harvesting devices [52]. Some comments should made regarding plasmonic applications of semiconductors. Extensive studies of permittivity spectra in optical frequencies for several semiconductors have been performed by Aspnes and Studna [53] at wavelengths λ > 206 nm. The semiconductors studied in their paper exhibit negative real permittivity values for λ < 310 nm (E=hc/λ > 4 eV) and possess relatively large dielectric losses, as shown in Table 1. The upper limits of the wavelength in Table 1 were limited to such values that are useful for the fabrication of bulk plasmonic resonators, i.e., $\varepsilon_{r}^{\prime }>$ −10. The lower limits of the wavelength for semiconductors are related to the data availability [53] and for metals to −1 ${>\varepsilon }_{r}^{\prime }>$ −10. Although plasmonic resonances may appear for $\varepsilon_{r}^{\prime }<$ −10 in larger samples, in such cases, the radiation losses are too large for their practical use (compare Figure 5a).

As it is seen in Table 1, plasmonic resonances in samples made of semiconductors should appear in the ultraviolet spectrum, and they would exhibit relatively low Q-factors. However, as shown in [53], in red and infrared wavelength spectra, two of the studied semiconductors, namely Si and GaP, exhibit very low dielectric losses, and their real part of permittivity is on the order of 10. Materials with such properties can be used for the construction of whispering gallery mode resonators. The resonance frequencies of whispering gallery mode resonators are size dependent [24]. Q-factors due to radiation losses for spherical ${TE}_{n01}$ modes (magnetic dipole and magnetic multipoles) of whispering gallery mode resonators increase with the mode index *n* [24]. For *n* = 1, 2, and 3 and the real permittivity $\varepsilon_{r}$ = 10, the Q-factors due to radiation losses would be $Q_{r}$ = 9.1, 32, and 117, respectively [24]. The diameter of a spherical ${TE}_{301}$ mode whispering gallery mode resonator made of Si or GaP operating at a wavelength of 780 nm would be about 440 nm. It should be noted that meanings and the electromagnetic properties of the magnetic dipole (magnetic multipole) whispering gallery mode resonators are distinct from those of plasmonic magnetic dipole (magnetic multipole) resonators. 

## 6. Comments and Future Directions

Materials that exhibit negative permittivity or permeability are encountered in different areas of physics and engineering, such as plasmonics, atmospheric physics, microwave electronics, and the physics of superconductors. Such materials have unique electromagnetic properties that are especially manifested in plasmonic resonators that are made of them. Each plasmonic resonance is associated with a specific negative value of permittivity (permeability) that generally depends on the size and shape of the sample and on the mode of oscillations. Sample shape dependency of the plasmonic resonance frequency is important and generally can be analyzed with advanced electromagnetic simulators [8]; however, regardless of the shape, the permittivity (permeability) related to the resonance appearance is always negative (although sometimes very close to 0) [38]. Plasmonic resonators typically have a size (at least one of its dimensions) that is much smaller than the wavelength. Radiation losses for open subwavelength plasmonic resonators are smaller than those for open dielectric resonators, especially when d/λ << 1; however, they should not be too small to ensure sufficient coupling with the incident radiation (Figure 5c). As it has been shown in this paper based on the grounds of electrodynamic analysis, in many aspects, the physical properties of electric and magnetic plasmonic resonators are similar. For an ionosphere subjected to the static magnetic field of the Earth, $\omega_{B}$ values become on the same order as $\omega_{p}$, and the properties of electric plasmons are similar to the magnetic plasmons described in this paper. Rigorous electrodynamic analysis of plasmonic resonators explains several physical phenomena that are experimentally observed by researchers working on various plasmonic resonators but also on atmospheric physics.

## Figures and Tables

**Table 1:** The real and the imaginary parts of the permittivity for semiconductors and metals in the range of wavelengths where they can be used as plasmonic resonators.

Material	λ (nm)	*E* (eV)	$\varepsilon_{r}^{\prime }$	$\varepsilon_{r}^{\prime \prime }$
Au [11]	582	2.13	−8.10	1.66
	368	3.37	−1.40	5.61
Ag [11]	471	2.63	−8.23	0.29
	332	3.74	−1.29	0.32
Si [53]	243	5.10	−9.29	10.77
	206	6.00	−7.44	5.88
Ge [53]	253	4.90	−9.11	9.59
	206	6.00	−6.65	5.67
GaP [53]	225	5.50	−10.27	10.97
	206	6.00	−5.52	7.04
GaAs [53]	238	5.20	−9.58	11.14
	206	6.00	−4.51	6.25
InSb [53]	310	4.00	−6.72	19.44
	206	6.00	−3.84	3.68
